# The protective effect of maternal electroacupuncture on prenatal nicotine exposure-induced intrauterine growth restriction in rats by improving placental angiogenesis

**DOI:** 10.1186/s13020-026-01331-1

**Published:** 2026-01-23

**Authors:** Xiaoxuan Liu, Bo Ji, Yitian Liu, Liyu Liu, Yang Fang, Shiqi Guo, Ling Zhang, Tingting Guo, Reiko Sakurai, Virender K. Rehan

**Affiliations:** 1 School of Acupuncture-Moxibustion and Tuina, Beijing University of Chinese Medicine, Beijing, 100029 China; 2 David Geffen School of Medicine at UCLA, Lundquist Institute for Biomedical Innovation at Harbor-UCLA Medical Center, Los Angeles, CA USA

**Keywords:** Electroacupuncture, IUGR, Placental angiogenesis, Placental perfusion

## Abstract

Fetal intrauterine growth restriction (IUGR) is a common pregnancy complication that significantly impacts fetal health and long-term outcomes. Prenatal nicotine exposure (PNE) is a major environmental risk factor for IUGR, with abnormal placental angiogenesis, leading to insufficient placental perfusion, which represents a key pathological process. Electroacupuncture (EA), a non-pharmacologic traditional Chinese medicine therapy, is known to regulate qi and blood flow and improve circulation. This study investigated whether EA could reverse PNE-induced IUGR by enhancing placental angiogenesis and explored the underlying mechanisms. In a PNE-induced IUGR rat model, daily EA treatment was applied at bilateral “ST36” acupoints. On gestational day 20, fetal and placental growth parameters, along with placental perfusion, were assessed. Placental RNA sequencing (RNA-seq) was performed to identify relevant biological pathways, with key pathway molecules validated by qRT-PCR and Western blot. EA significantly restored fetal weight and length and increased placental weight and diameter. It also reduced the umbilical artery resistance index and improved placental perfusion. Furthermore, EA increased placental vascular density. Bulk RNA-seq revealed EA induced substantial changes in placental gene expression, including significant upregulation of the key angiogenic factor placental growth factor (PGF). Gene Ontology (GO) enrichment analysis indicated that differentially expressed genes were primarily involved in stress response regulation and cell surface receptor-mediated signal transduction, with notable enrichment in the PI3K/AKT signaling pathway. These transcriptomic findings were validated by qRT-PCR and Western blot, which confirmed that EA upregulated the mRNA expression of PGF, VEGFR-1, PI3K, and AKT, and increased the protein levels of PGF, VEGFR-1, and the phosphorylation of PI3K/AKT (p-PI3K, p-AKT). This integrated evidence suggests that maternal EA treatment may promote placental angiogenesis via activation of the PGF/VEGFR-1/PI3K/AKT pathway, thereby protecting against PNE-induced IUGR.

## Introduction

Intrauterine growth restriction (IUGR) is a common complication of pregnancy defined as the inability of the fetus to attain its genetic growth potential in utero [1]. A recent multinational cohort study in *The Lancet* reported that 17.4% of babies are born with IUGR [2]. IUGR is associated with multiple adverse outcomes, including premature birth, neonatal respiratory distress syndrome, fetal pulmonary hypoplasia, and impaired neurodevelopment [3, 4]. In severe cases, it even causes perinatal death, accounting for 26.6% of all neonatal deaths, representing the second-leading cause of perinatal mortality [5]. Tobacco exposure during gestation is one of the significant triggering factors for IUGR in offspring [6]. Globally, 1.7% of pregnant women actively smoke, while in low- and middle-income countries, up to 73% of pregnant women are exposed to second-hand smoke [7, 8]. Prenatal nicotine exposure (PNE)-induced IUGR is closely related to placental hypoperfusion. As the vital organ connecting the mother and fetus, the placenta effectively transports nutrients and oxygen to the fetus and removes metabolic wastes from the fetal body through its rich blood flow, thereby maintaining normal pregnancy and fetal growth and development [9, 10]. When the placental perfusion is insufficient, it can limit the supply of nutritional oxygen to the fetus, ultimately leading to IUGR [11]. Studies have shown that PNE can cause a decrease in the volume, surface area, and length of placental capillaries [12], thereby reducing placental perfusion [13] and ultimately resulting in IUGR. Multiple signaling pathways intricately regulate placental perfusion, including VEGF, HIF, ERK, PI3K/AKT, Notch, NO, RAS, COX [14–17]. However, the mechanism by which PNE causes structural abnormalities in placental capillaries and subsequent hypoperfusion is still unclear.

According to *Fetal Growth Restriction: ACOG Practice Bulletin*, apart from careful screening and evaluation during pregnancy, there is no effective way to prevent fetal growth restriction. For certain cases, antenatal glucocorticoids are administered to accelerate fetal lung maturation, while magnesium sulfate is utilized for fetal and neonatal neuroprotection [18]. However, excessive antenatal steroids can suppress fetal development [19] and magnesium sulphate may cause feeding intolerance [20]. We previously demonstrated that EA at the “ST36” acupoints in maternal rats exposed to perinatal nicotine could effectively protect their offspring from IUGR [21, 22]. Furthermore, we discovered that combined antenatal and postnatal EA administration yielded more pronounced therapeutic effects than postnatal intervention alone, indicating that EA exerts its protective actions during pregnancy. However, it is unclear how maternal EA protects offspring against IUGR. Given the central role of placental hypoperfusion in PNE-induced IUGR and EA’s established ability to enhance angiogenesis and perfusion in multiple organs (uterus, brain, heart, liver, kidneys) [23–27], we hypothesized that EA mitigates PNE-induced IUGR by improving placental perfusion. Therefore, this study investigated whether EA could reverse PNE-induced IUGR by enhancing placental angiogenesis and explored the underlying mechanisms.

## Materials and methods

### Animals and ethics

Eighteen Specific Pathogen Free (SPF)-grade healthy female SD rats (9 weeks old, 280–320 g) and six SPF-grade healthy male SD rats (10 weeks old, 300–340 g) were obtained from Beijing Vital River Laboratory Animal Technology Co., Ltd. (Certificate number: SCXK [Beijing] 2021-0011). Animals were housed at a room temperature of 24 ± 1 °C and relative humidity of 55 ± 5%, under a 12 h light/12 h dark cycle. They were provided standard chow and water ad libitum for one week before the experiments. All experimental procedures involving animals were performed in strict compliance with the 3Rs principle (Replacement, Reduction, Refinement) and approved by the Animal Ethics Committee of Beijing University of Chinese Medicine (Approval No. BUCM-2024120403-4278).

### Experimental protocol

**Fig. 1:**
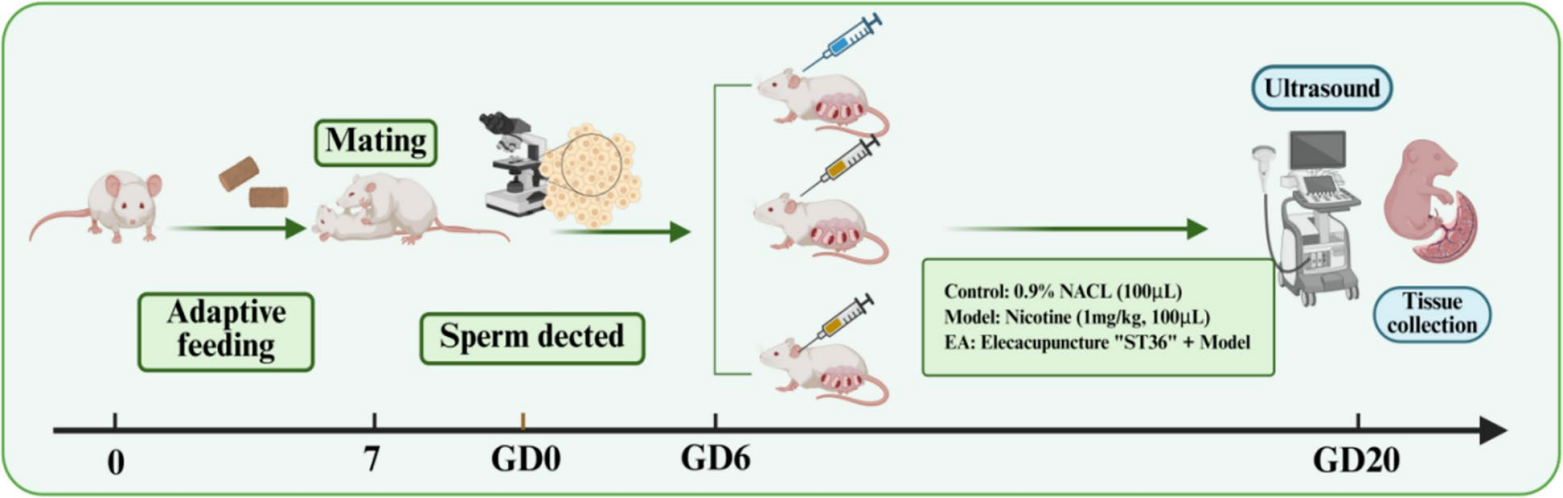
Workflow diagram. Showing the establishment of the IUGR model and interventions

Figure 1 shows the establishment of the IUGR model and interventions. After the acclimatization period of one week, eighteen female rats were randomly divided into Control group, Model group, and EA group by the random number table method, with 6 rats in each group. Male and female rats mated at night in a 1:3 ratio, and the vaginal smears were taken the next morning to observe sperm under an optical microscope. The day the sperm was found is recorded as day 0 of pregnancy. The preparation method for the IUGR model in References [28, 29]. In the Model group, nicotine (1 mg/kg, 100 μL prepared with 0.9% sodium chloride solution) was injected subcutaneously into the dorsal cervical region of rats from the 6th day of gestation, once a day until the 19th day of gestation. Rats in the Control group were injected subcutaneously into dorsal cervical region of rats with 0.9% sodium chloride solution at the same time. The EA group was administered EA along with nicotine administration, as described in the Model group. On gestational day 20, placental perfusion was assessed by color Doppler ultrasound. The animals were then sacrificed, and samples were collected. Fetal weight, crown-rump length, placental weight, and placental diameter were measured and recorded. Part of the placenta was fixed in 4% paraformaldehyde for subsequent hematology and eosin (H&E) staining, and the rest was stored in the − 80 °C refrigerator for subsequent molecular testing.

### Electroacupuncture treatment

The hind limbs of the female rats in the EA group were fixed, and the acupoints of “ST36” were selected according to the “Science of Experimental Acupuncture and Moxibustion”. The points of “ST36” were located at the posterolateral knee joint of the hind limbs of the rats, about 5 mm below the small head of the fibula. From gestational day 6 to day 19, acupuncture needles (0.2 mm × 13 mm, Hwato) were inserted vertically into the bilateral ST36 acupoints to a depth of 7 mm and connected to the negative pole. The positive pole of EA was connected to a needle inserted horizontally into the skin, 2 mm below ST36. The EA parameters were as follows: frequency 2/15 Hz, intensity 1 mA, duration 20 min, and administered once a day between 10 AM and Noon.

### Color Doppler ultrasound

On the 20th day of gestation, umbilical artery hemodynamics were assessed using the Vevo2100 ultra-high frequency high-resolution ultrasound system (VisualSonics, US) under sodium pentobarbital anesthesia (45 mg/kg, intraperitoneal injection). Following abdominal fur removal and acoustic gel application, the ultra-high frequency electronic linear array probe was positioned on the abdomen for imaging. The locations of the placenta, umbilical artery, and umbilical vein were identified based on the acquired images. For each female rat, the blood flow spectra of the umbilical arteries from three randomly selected fetuses were collected. Subsequently, Pulsatility Index (PI = [PSV—EDV]/TAV), Resistance Index (RI = [PSV—EDV]/PSV), and Systolic-Diastolic ratio (S/D) of the umbilical artery were calculated from Doppler wave forms, where PSV denotes peak systolic velocity, EDV denotes end-diastolic velocity, and TAV denotes time-averaged velocity.

### H&E staining of placenta

Placenta tissue samples from each group were fixed in paraformaldehyde for 48 h, dehydrated in ethanol, embedded in paraffin, and then cut into 4-μm sections along the circular cross-section of the placenta at the same thickness. Six randomly selected placentas from each group were evaluated. After deparaffinization and hydration, sections were stained with H&E. The morphology of placental tissue was observed under 4 × and 20 × magnifications using TX5 microscope (Olympus, Japan). The area of labyrinth zone (LZ) was measured using ImageJ software. Finally, vascular density was calculated as the number of vessels per mm^2^ of the LZ.

### Bulk RNA-seq of placenta

#### RNA extraction

Four frozen placental tissues were taken from each group for processing and sequencing. Total RNA was extracted using TRIzol^®^ Reagent. Then RNA quality was determined by the 5300 Bioanalyser (Agilent) and quantified using the ND-2000 (NanoDrop Technologies). Only high-quality RNA samples (OD260/280 = 1.8 ~ 2.2, OD260/230 ≥ 2.0, RQN ≥ 6.5, 28S: 18S ≥ 1.0, > 1 μg) were used to construct sequencing libraries.

### Library preparation and sequencing

The placental RNA-seq transcriptome library was prepared using Illumina^®^ Stranded mRNA Prep, Ligation (catalog#: 20040525, San Diego, CA), using 1 μg of total RNA. Messenger RNA was isolated according to polyA selection method by oligo (dT) beads and then fragmented. Then double-stranded cDNA was synthesized using a SuperScript double-stranded cDNA synthesis kit (Invitrogen, CA) with random hexamer primers. The synthesized cDNA was subjected to end-repair, phosphorylation, and adapter addition according to the library construction protocol. Libraries were size-selected for 300 bp fragments via 2% Low Range Ultra Agarose electrophoresis. Amplification was performed using Phusion DNA polymerase (NEB) for 15 PCR cycles. After quantification by Qubit 4.0, the sequencing was performed on NovaSeq X Plus platform (PE150) using NovaSeq Reagent Kit.

### Quality control and read mapping

The raw paired-end reads were trimmed and quality controlled by fastp with default parameters. Then, clean reads were separately aligned to the reference genome with orientation mode using HISAT2 software. The mapped reads of each sample were assembled by StringTie in a reference-based approach.

### Differential expression analysis and functional enrichment

Differentially expressed genes (DEGs) were identified through transcript quantification using the transcripts per million reads (TPM) method. Gene abundance was quantified via RSEM, followed by differential expression analysis with DESeq2. DEGs with |log_2(_Fold Change)|≥ 0.585, and P-value < 0.05 were considered to be significantly DEGs. In addition, functional-enrichment analysis, including Gene Ontology (GO) and Kyoto Encyclopedia of Genes and Genomes (KEGG), was performed to identify which DEGs were significantly enriched in GO terms and metabolic pathways at Bonferroni-corrected P-value < 0.05 compared with the whole-transcriptome background. GO functional enrichment and KEGG pathway analysis were carried out by Goatools and Python scipy software, respectively.

### qRT-PCR

As described above, total RNA was extracted from the placental tissue using TRIzol^®^ Reagent. RNA concentration and purity were measured using the ultraviolet spectrophotometer. Total RNA was reverse-transcribed into single-stranded cDNA using the RevertAid First Strand cDNA kit (Thermo Fisher Scientific) at 42 ℃ for 60 min, 70 ℃ for 5 min in a total volume of 20μL. The PCR reaction mix consisted of 1μL cDNA, 10μL Power SYBR^®^ Green PCR Master Mix (Thermo Fisher Scientific), 1μL each of forward and reverse primers, and 7μL Nuclease-free water. All primers were synthesized by Sangon Biotech (Shanghai) Co., Ltd.

The primers listed were amplified (Fig. 7). The reactions proceeded by an initial denaturation step at 95 ℃ for 2 min, followed by 40 repeated thermal cycles (95 ℃ for 30 s, 60 ℃ for 30 s). Relative gene expression normalized to GAPDH was calculated using the 2^−∆∆CT^ method.

### Western blot

The reagents were provided by Wuhan Servicebio Biotechnology Co., Ltd. (Servicebio Biotech, Wuhan, China). The placental tissue was homogenized in RIPA lysate buffer for total protein extraction, and its concentration was determined by BCA method. Equal amounts of protein were separated by SDS-PAGE electrophoresis and then transferred onto PVDF membranes. After transferring, the membranes were rapidly washed in TBST buffer once, incubated at room temperature for 30 min in 5% skimmed milk solution. Primary antibodies (PGF, VEGFR-1, PI3K, p-PI3K, AKT, and p-AKT) were incubated overnight at 4 °C with continuous shaking, all at a 1:1000 dilution. Following three 5-miniute TBST washes, membranes were incubated with HRP-conjugated secondary antibody (1:5000) for 30 min at room temperature. Protein bands were visualized by ECL chemiluminescence, with band intensities quantified using Image Lab 6.0 software (Bio-Rad) and normalized to β-actin (1:5000) loading control.

### Statistical analysis

All statistical tests were run using R4.2.2, and all graphics are plotted using BioRender (https://biorender.com/). The results are presented as mean ± standard deviation (SD). For normally distributed data with homogeneous variance, intergroup differences were analyzed by one-way ANOVA with Bonferroni test; for data with unequal variance, Welch’s ANOVA was applied; for small-sized non-normally distributed data, the Kruskal–Wallis test was utilized. p < 0.05 was considered statistically significant.

## Results

### Fetal and placental developmental parameters

**Fig. 2:**
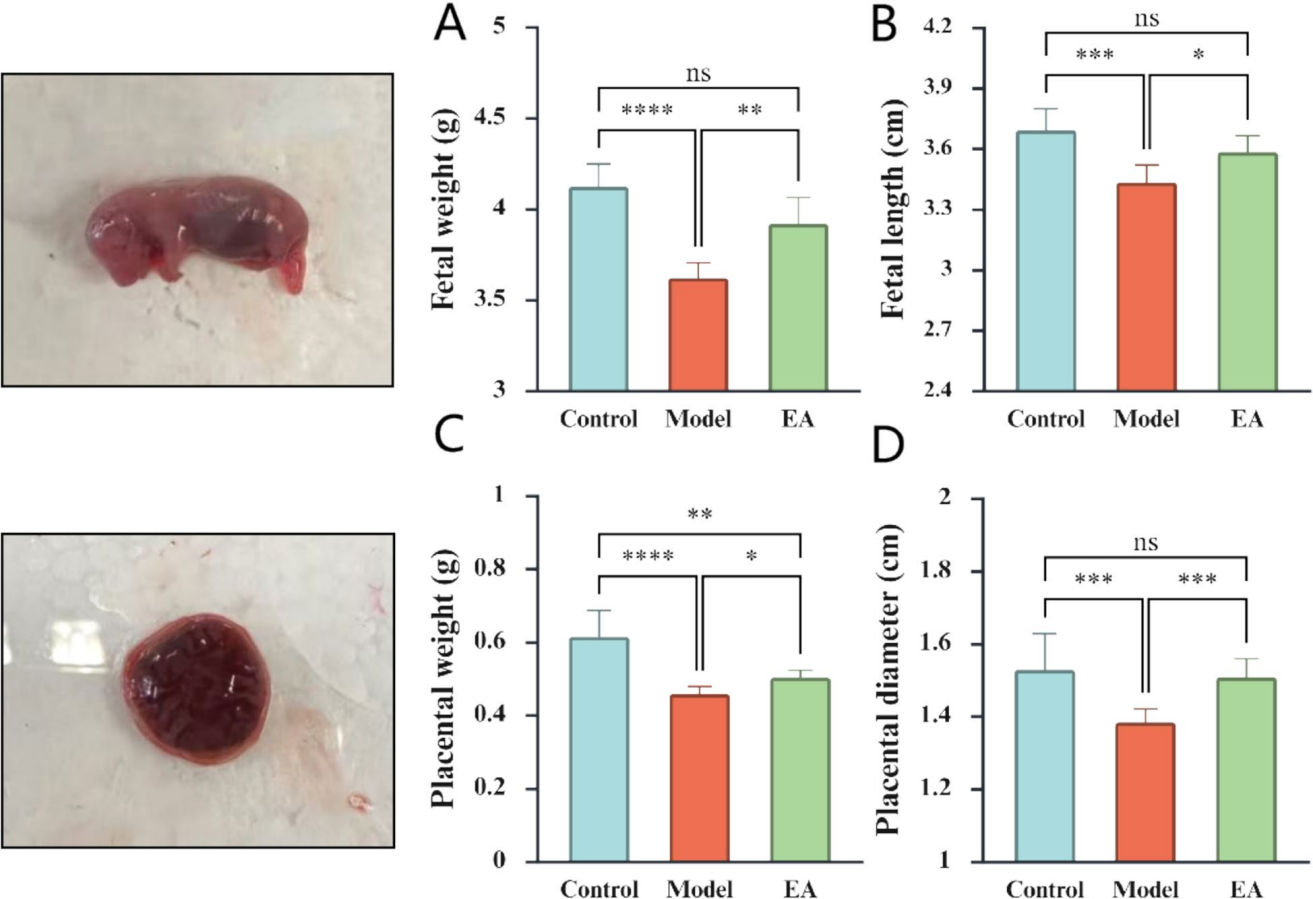
Fetal and placental developmental parameters (n = 12). Fetal weight (**A**), length (**B**), placental weight (**C**), and diameter (**D**) in the Control, Model, and EA groups at 20 days of gestation. *p < 0.05, **p < 0.01, ***p < 0.001, **** p < 0.0001, ns indicates no significance

The results demonstrate that, on the 20th day of gestation, the fetal weight, crown-rump length, placental weight, and placental diameter in the Model group were significantly reduced compared to those in the Control group. In contrast, the above parameters were normalized in EA group (Fig. 2).

### Color Doppler ultrasound

**Fig. 3:**
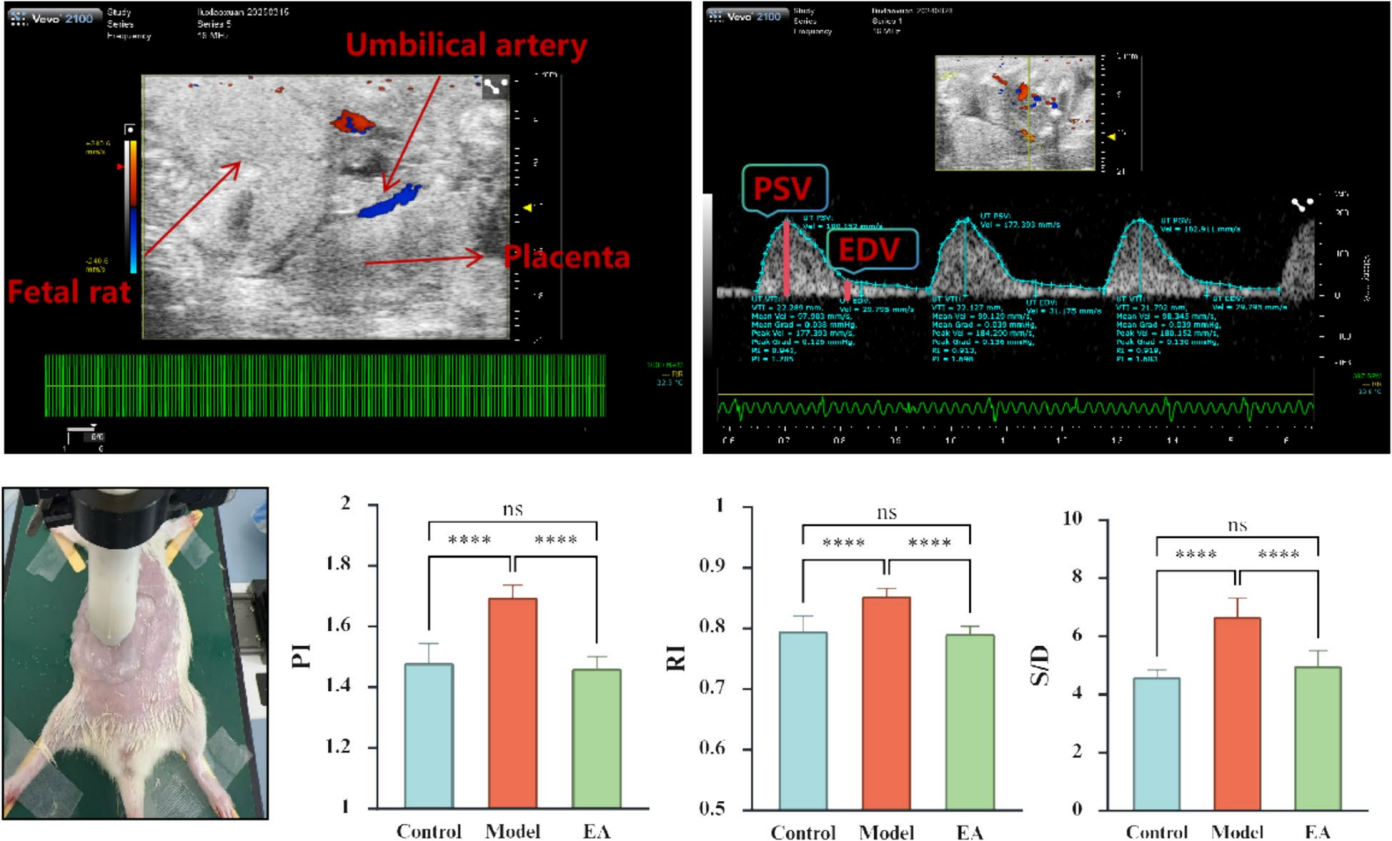
Measured and calculated Doppler ultrasonography indices (n = 12). *PSV* peak systolic velocity, *EDV* end-diastolic velocity, *RI* resistive index, *PI* pulsatility index, *S/D* systolic-to-diastolic ratio, *TAV* time-averaged peak velocity (used to calculate PI but not used as an individual measurement). *p < 0.05, **p < 0.01, ***p < 0.001, **** p < 0.0001, ns indicates no significance

The results show that, compared with the Control group, the values of S/D, PI and RI are significantly increased in the Model group, whereas they are significantly decreased in the EA group compared with the Model group (Fig. 3). These hemodynamic changes indicate that PNE reduces placental perfusion, while EA restores it.

### Placental morphology

**Fig. 4:**
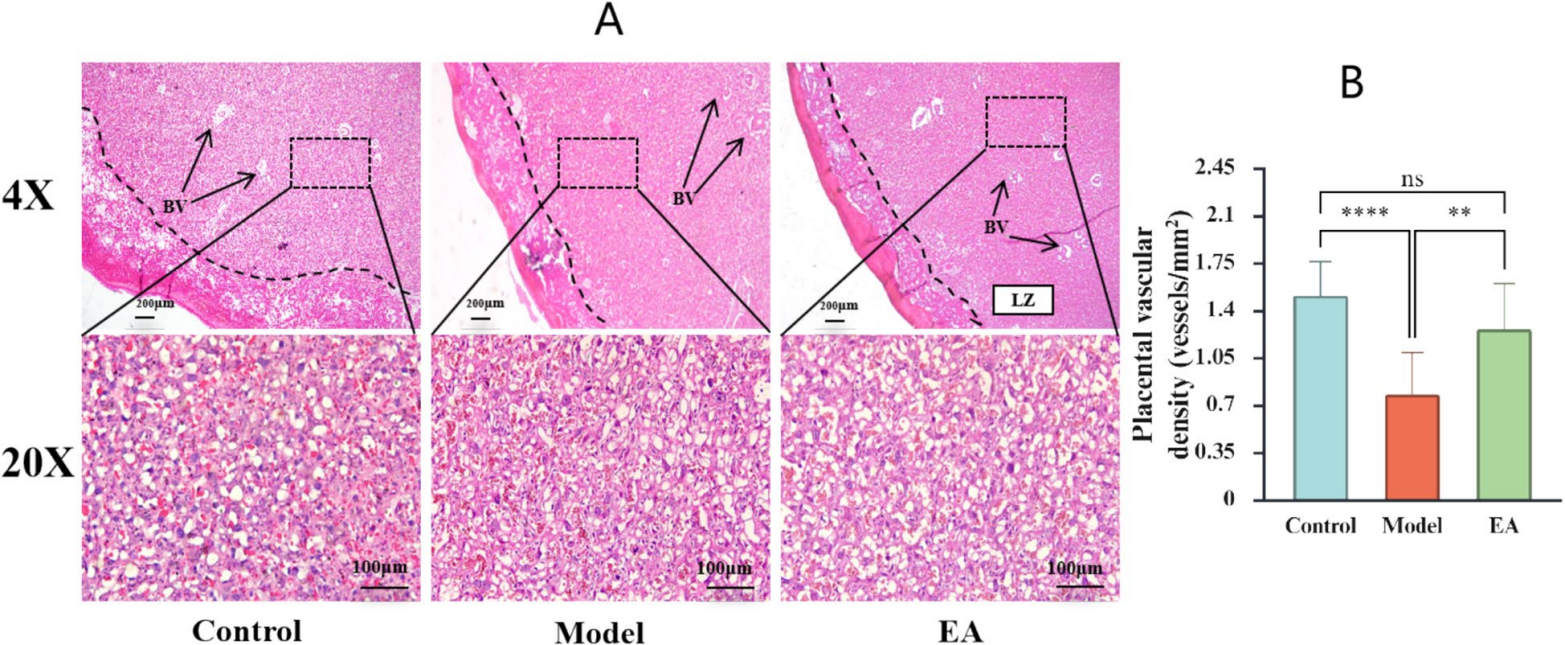
Placental Morphology and vascular density. **A** Representative images of H&E-stained placenta tissue sections used in histomorphological analysis in the Control, Model, and EA groups. **B** Placental vascular density (n = 12). *p < 0.05, **p < 0.01, ***p < 0.001, **** p < 0.0001, ns indicates no significance

Normal placental architecture is stratified into three distinct morphological and functional layers from the fetal side to the maternal side: the labyrinth, basal, and decidua. The LZ consists of a complex, web-like vascular network comprising sinusoids for maternal blood circulation and capillaries for fetal circulation. These two circulatory systems are closely intertwined yet separated by vascular membranes composed of cytotrophoblast and syncytiotrophoblast cells. Our H&E staining results show that compared with the Control group, the Model group exhibited ruptured and shrunken placental septa, decreased areas of blood sinusoids irregular morphology, and nuclear shrinkage. Compared with the Model group, these pathological changes were improved in the EA group. These findings suggest that nicotine damages the vascular structure of the placental labyrinth, and that EA mitigates these changes (Fig. 4A).

### Placental vascular density

To investigate whether the vasculature of the placenta was affected by EA, the vascular density of the labyrinth area was assessed. A significant decrease in vascular density was observed in the Model group compared to the Control group, whereas the EA group showed a significant increase compared to the Model group (Fig. 4B).

### Bulk RNA-seq results of the placenta from rat fetuses in each group

#### Analysis results of quality control

**Fig. 5:**
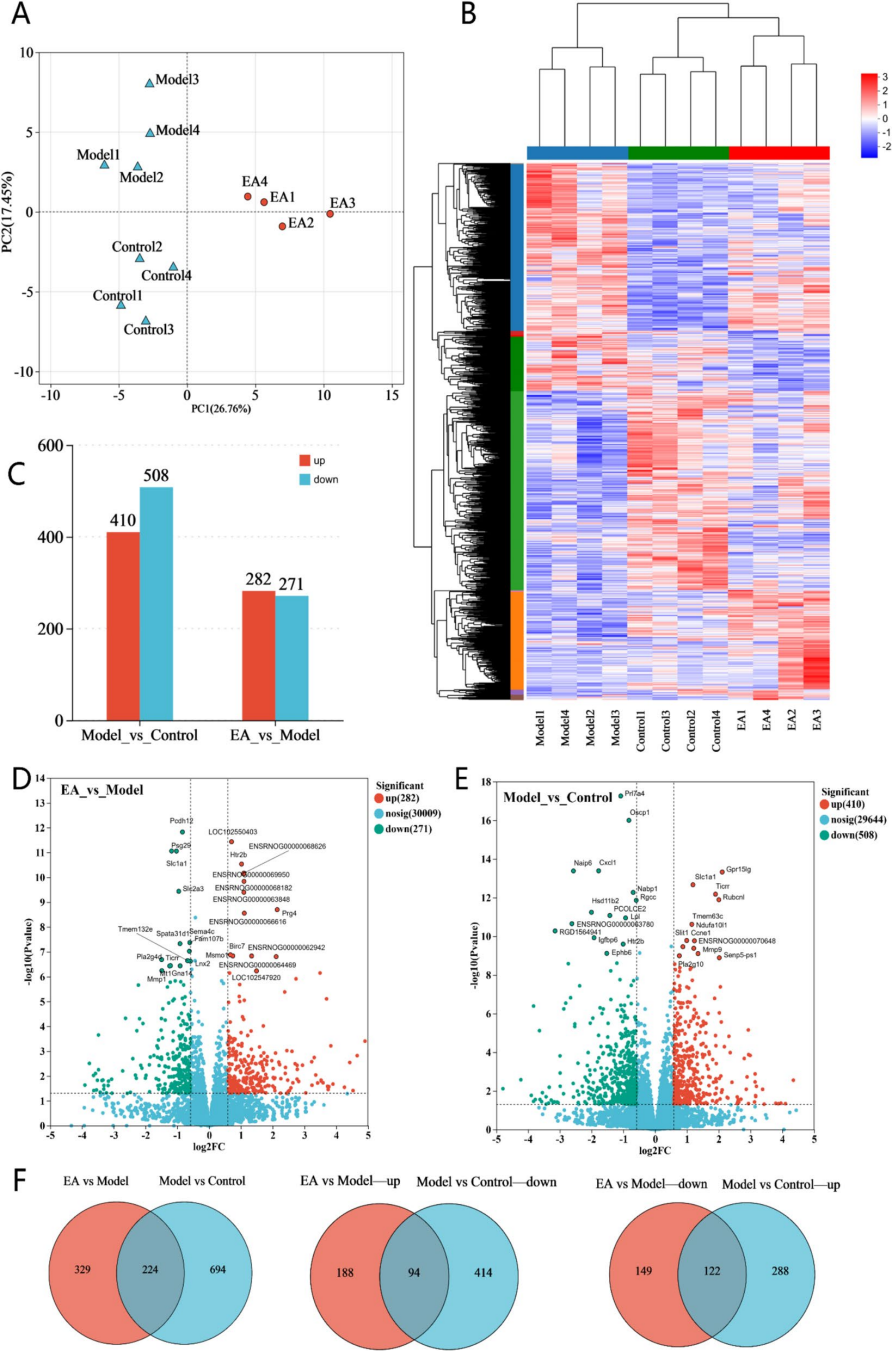
Overview of bulk RNA-seq sequence alignment and data dispersion. **A** Principal Component Analysis. The distances between each sample point represent the distance between the samples. The closer the distances are, the higher the similarity between the samples indicates, which means the biological reproducibility of the samples is better. **B** Hierarchical clustering heatmap of DEGs. (n = 4). This heatmap can visually compare the homogeneity and heterogeneity among different groups. Each column in the figure represents a sample, and each row represents a gene. The color in the figure indicates the expression level of the gene in the sample; red represents high expression, and blue represents low expression. The numerical labels next to the color bar in the upper left corner represent the specific trend of expression levels. On the left side, there is a dendrogram of gene clustering and a module diagram of sub-clustering. The closer the two gene branches are, the more similar the expressions are. The upper figure is a tree diagram of sample clustering, and the lower figure shows the names of the samples. The closer the two sample branches are, the more similar the expression patterns of all genes in the two samples are. **C** Bar expression plots of differentially expressed genes among each group. **D–E** Volcano plot of DEGs. The X-axis represents log2FoldChange, and the Y-axis represents-log10 (p-value). **F** Venn diagram of DEGs. Circles of different colors represent different gene sets, and the values represent the common and specific genes among different gene sets

To ensure the quality of the original sequencing data, it is necessary to evaluate the quality of the original data before analysis. The results showed that the clean bases of each sample all reached more than 5.99 GB, and the percentage of Q30 bases was more than 95.49%. The Clean Reads of each sample were aligned with the designated reference genome, and the alignment rates ranged from 97.62% to 97.89%, indicating that the sequencing results were reliable (Table 1). The PCA plot showed that the sequencing samples within groups are similar and comparable (Fig. 5A).

**Table 1:**
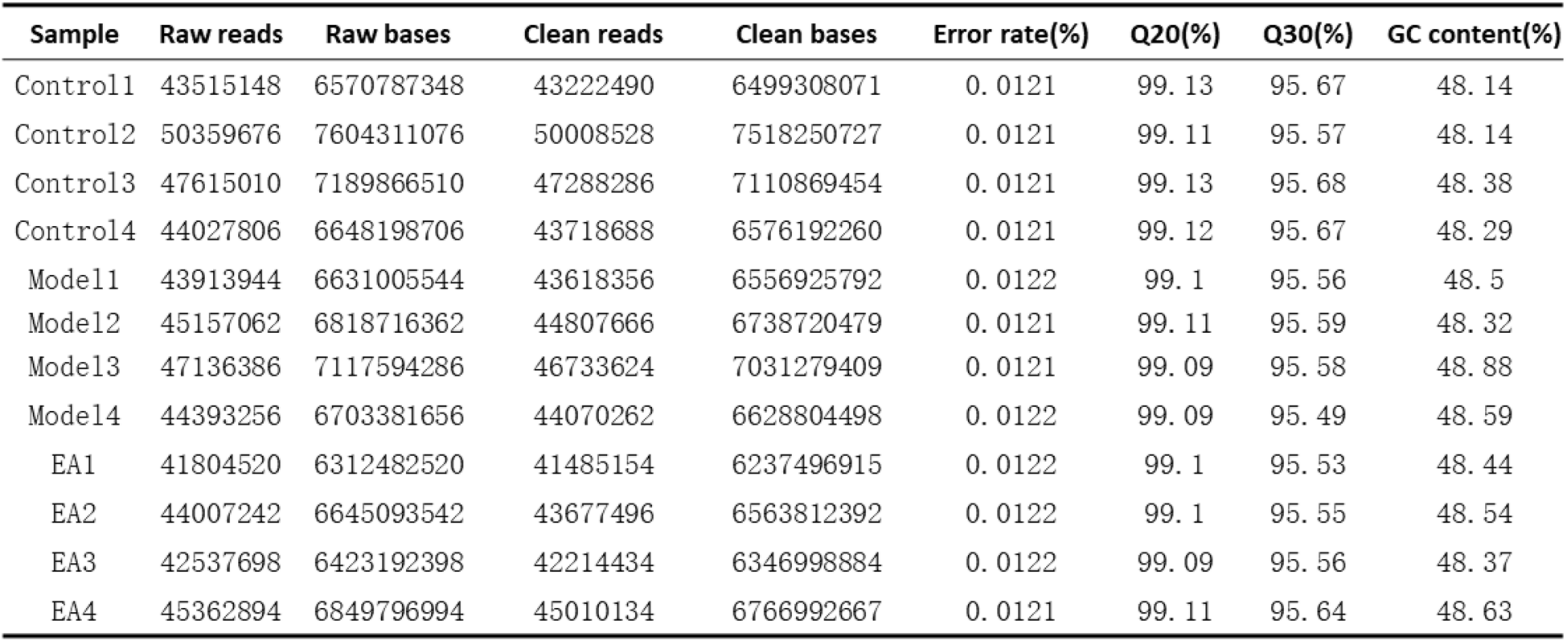
Quality control and sequencing information for samples (n = 4)

(1) Sample: Sample name; (2) Raw reads: The total number of entries of the original sequencing data; (3) Raw bases: The total amount of original sequencing data (the number of Raw reads multiplied by the read length); (4) Clean reads: The total number of sequencing data entries after quality control; (5) Clean bases: The total amount of data sequenced after quality control (the number of Clean reads multiplied by the length of reads); (6) Error rate (%): The average error rate of sequencing bases corresponding to the quality control data, which is generally below 0.1%; (7) Q20 (%), Q30 (%): Quality assessment is conducted on the sequencing data after quality control. Q20 and Q30, respectively, refer to the percentages of bases with sequencing quality above 99% and 99.9% of the total bases. Generally, Q20 is above 85% and Q30 is above 80%; (8) GC content (%): The percentage of the total G and C bases corresponding to the quality control data in the total bases.

#### Differential gene expression results

When the threshold was set at “|log_2(_Fold Change)|≥ 0.585, and p < 0.05”, a large number of DEGs were identified in each pairwise comparison. The clustering heatmap of DEGs showed that gene expression within each group was relatively consistent (Fig. 5B). Compared with the EA and Control groups, the Model group displayed a distinctly different color pattern, indicating marked separation of gene expression. In contrast, the EA group closely resembled the Control group, suggesting a similar gene expression profile. The volcano plot and column chart revealed 918 DEGs between the Model and Control groups (410 upregulated, 508 downregulated) and 553 DEGs between the EA and Model groups (282 upregulated, 271 downregulated) (Fig. 5C–E). Notably, 224 genes were differentially expressed in both comparisons: 122 genes were upregulated in the Model/Control comparison but downregulated in the EA/Model comparison, while 94 genes were downregulated in Model/Control but upregulated in EA/Model. These findings suggest that EA may block the PNE-induced dysregulation of these genes to help regulate placental function (Fig. 5F).

#### Analysis results of gene ontology and Kyoto encyclopedia of genes and genomes

**Fig. 6:**
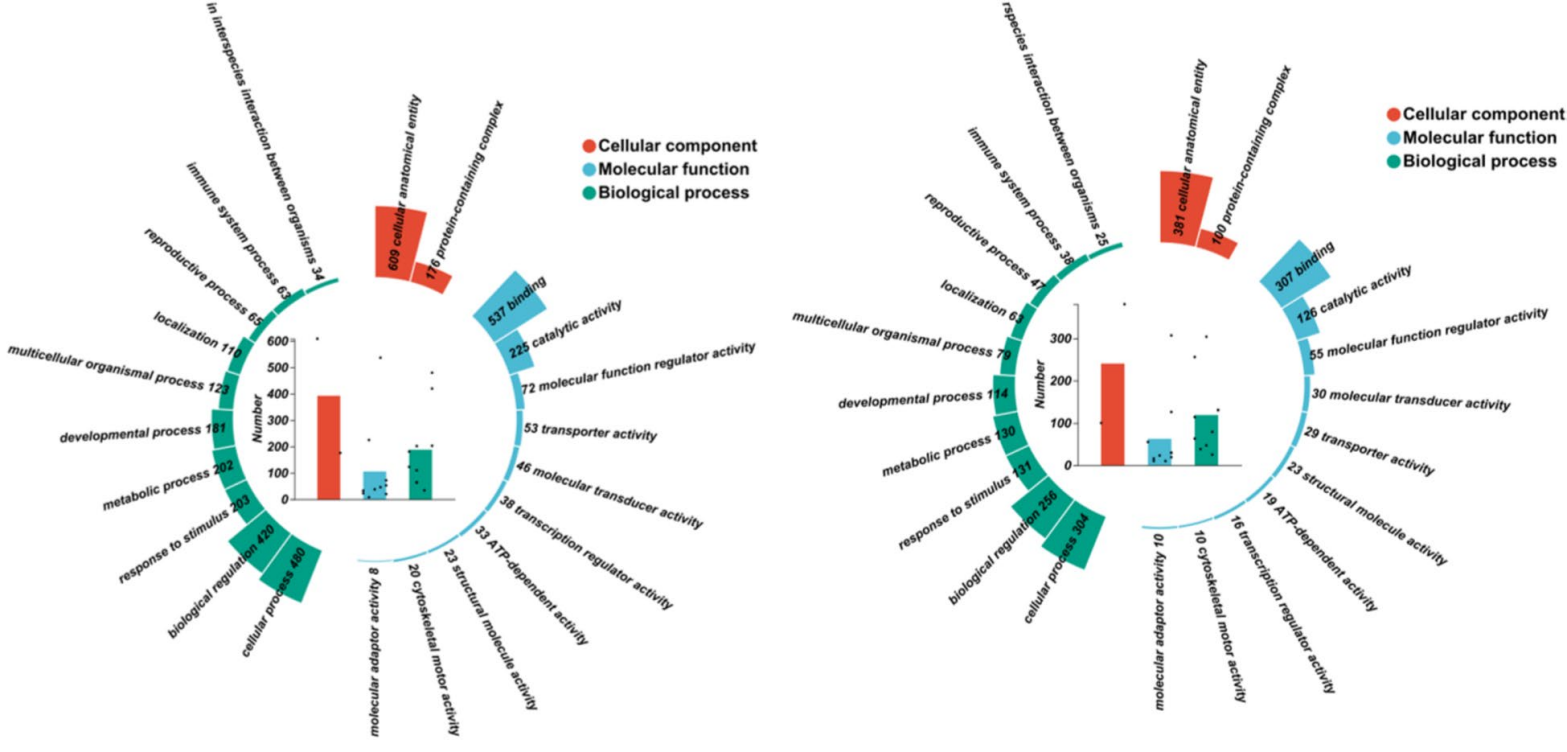
The GO functional annotation analysis results of bulk RNA-seq. The annular heat map on the outer circle shows the number corresponding to each secondary category under the primary category, while the central scatter bar chart represents the mean ± SD of the number of secondary categories in different primary categories

**Fig. 7:**
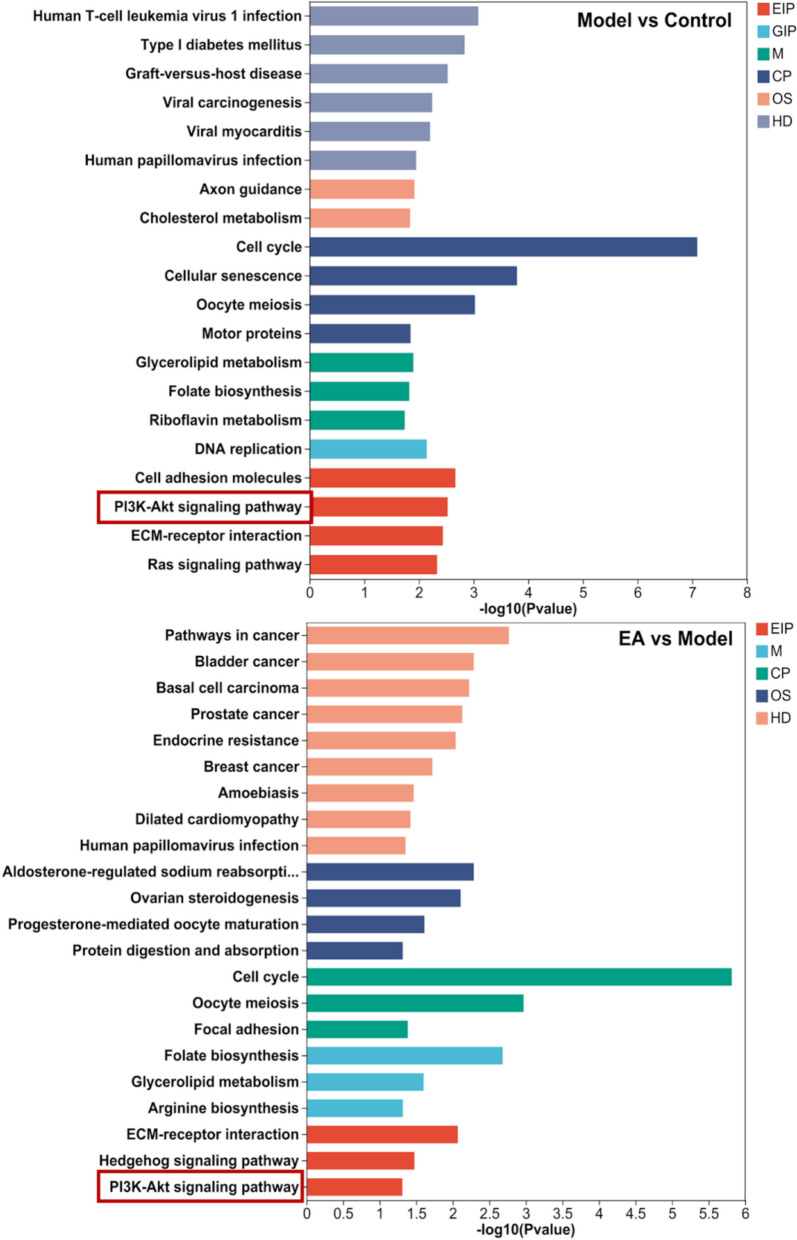
KEGG pathway enrichment results of Bulk RNA-seq. The Y axis represents the KEGG pathway, and the X axis represents the significance level of enrichment, corresponding to the height of the column. The smaller the p-value is, the larger the value of -log10 (p-value) will be. and the more significantly enriched the KEGG pathway is. Different colors represent the 7 branches of the KEGG metabolic pathway, namely metabolism (M), genetic information processing (GIP), environmental information processing (EIP), cellular process (CP), organism system (OS), human disease (HD), and drug development (DD)

To investigate the biological functions in the placenta tissue, GO annotations analysis was conducted on the identified DEGs. GO mainly includes three parts: biological process (BP), cell composition (CC), and molecular function (MF). GO terms are categorized into three domains: biological process (BP), cellular component (CC), and molecular function (MF) (Fig. 6). To explore the signaling pathways modulated by EA, KEGG pathway enrichment analysis was conducted. In the Model/Control comparison, DEGs were mainly enriched in “cell adhesion molecules,” “PI3K-Akt signaling pathway,” “ECM-receptor interaction,” and “Ras signaling pathway.” In the EA/Model comparison, enrichment was primarily observed in “ECM-receptor interaction,” “Hedgehog signaling pathway,” and “PI3K-Akt signaling pathway.” Notably, both comparisons shared enrichment in the “PI3K-Akt signaling pathway” and “ECM-receptor interaction” (Fig. 7).

### qRT-PCR

**Fig. 8:**
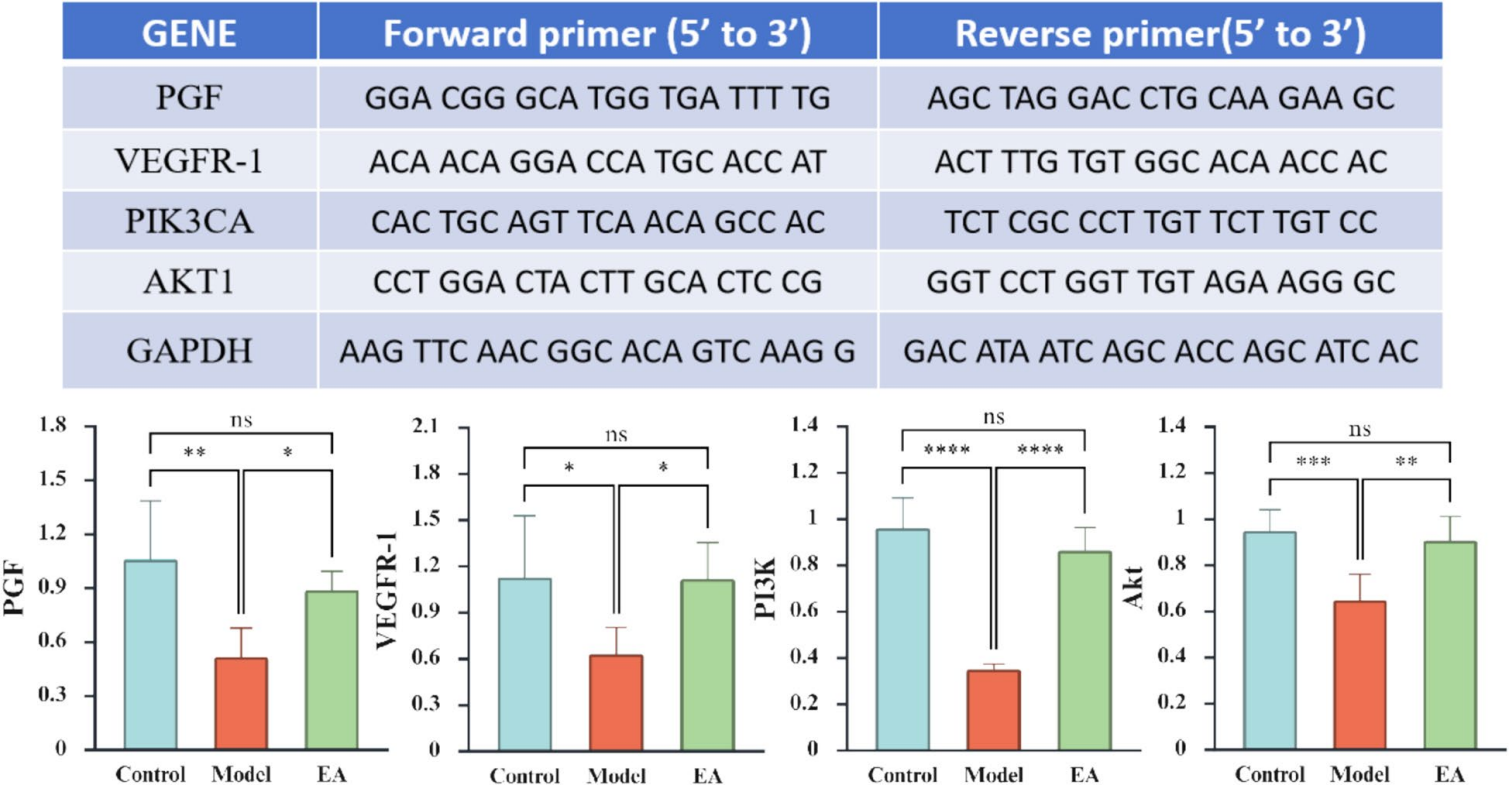
Results of qRT-PCR in each group (n = 6). The X-axis represents the grouping, and the Y-axis is the relative expression of the gene. *p < 0.05, **p < 0.01, ***p < 0.001, **** p < 0.0001, ns indicates no significance

Based on the results of RNA-seq analysis in placenta of rat fetuses in each group, and considering current knowledge of placental vasculature, we identified DEGs and key genes in pathways including Placental Growth factor (PGF), Vascular Endothelial Growth Factor Receptor 1 (VEGFR-1), Phosphatidylinositol 3-kinase (PI3K), and Protein Kinase B (AKT). qRT-PCR results showed that, compared with the Control group, the mRNA expression of PGF, VEGFR-1, PI3K, and AKT was significantly decreased in the Model group. In contrast, compared with the Model group, the expression of these genes was significantly increased in the EA group (Fig. 8). These findings suggest that EA may exert corrective effects on placental function by regulating the expression of PGF, VEGFR-1, PI3K, and AKT.

### Western blot

**Fig. 9:**
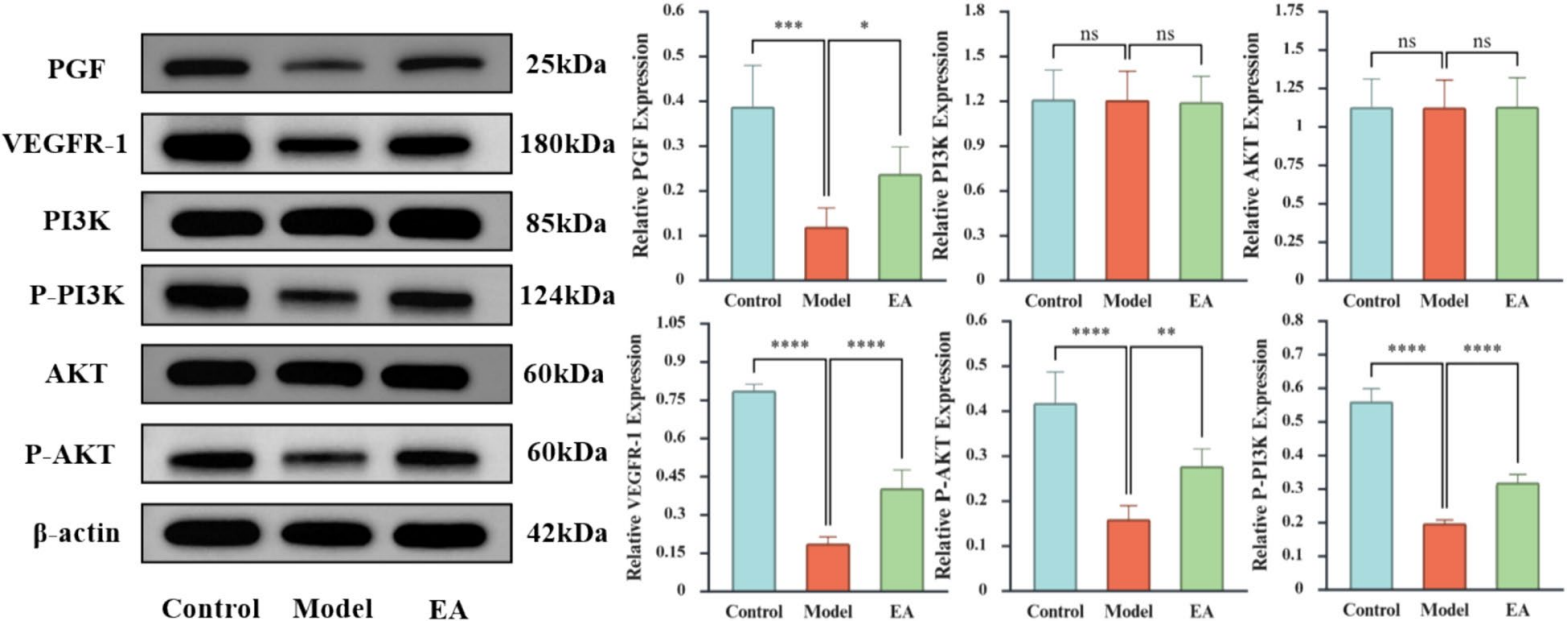
Results of Western blot in each group (n = 6). The X-axis represents the grouping, and the Y-axis is the relative expression of the protein. *p < 0.05, **p < 0.01, ***p < 0.001, **** p < 0.0001, ns indicates no significance

Compared with the Control group, the Model group showed significant downregulation of PGF, VEGFR-1, phosphorylated PI3K (p-PI3K), and phosphorylated AKT (p-AKT). In contrast, EA treatment markedly upregulated the expression and phosphorylation levels of these proteins relative to the Model group **(**Fig. 9**)**. These results indicate that EA may activate the PI3K/AKT pathway by enhancing PGF/VEGFR-1 signaling and promoting PI3K/AKT phosphorylation.

## Discussion

Impaired placental angiogenesis and consequent hypoperfusion constitute the core pathological insult underlying IUGR, which compromises fetal development and predisposes offspring to lifelong health sequelae [30, 31]. PNE directly disrupts placental angiogenesis and perfusion, thereby inducing IUGR [32–35] However, there is no safe and effective therapy. EA integrates traditional acupuncture and modern electrical stimulation techniques. By stimulating acupoints to activate bidirectional regulatory pathways, EA modulates neural signaling, endocrine hormone release, and immune factors balance, thereby treating various systemic diseases. ST36 plays a significant role in promoting the qi and blood in the human body. Multiple studies have confirmed that acupuncture at ST36 can effectively improve blood perfusion in multiple organs, including the uterus, stomach, brain, liver, and kidneys, and promote tissue repair. Our previous work demonstrated that EA at maternal ST36 acupoints alleviated IUGR and improved fetal organ development in the PNE rat model used in this study [21, 22] and the effect of EA initiated during pregnancy [36]. Therefore, using the placenta, the key organ connecting mother and fetus, as our focal point, we aimed to investigate the mechanism by which EA improves placental perfusion and prevents PNE-induced IUGR.

IUGR is characterized by low birth weight and stunting [1]. We observed a significant decrease in fetal weight and length of offspring in the Model group on the 20th day of gestation, aligning with prior findings [29, 37], confirming the successful establishment of an IUGR model induced by PNE. Remarkably, EA enhanced fetal weight and length, indicating its potential to ameliorate PNE-induced IUGR. Given the pivotal role of placental dysfunction in the pathogenesis of PNE-induced IUGR, placental weights and diameters were assessed. The results showed that EA significantly increased placental weight and diameter, suggesting a protective effect mediated through the amelioration of placental function.

Umbilical artery Doppler assessment is an important component in the diagnosis of IUGR, which can accurately reflect the status of placental perfusion. With fetal hypoxia and ischemia, umbilical arterial resistance is significantly increased [38]. Interestingly, both maternal smoking and IUGR are associated with increased umbilical artery resistance [33, 39], which is consistent with the results of findings in the current study. Moreover, we found that EA at the “ST36” acupoints in pregnant rats significantly reduced the RI, PI, and S/D values of the fetal umbilical artery, indicating that EA at the “ST36” point in pregnant rats can effectively reduce umbilical artery blood flow resistance in IUGR fetuses and increase placental perfusion.

Normal placental vascular formation and development are fundamental biological prerequisites for adequate placental perfusion and sufficient fetal blood oxygen supply. Conversely, Abnormal placental vascular development directly compromises materno-fetal exchange and represents a key pathological factor underlying IUGR [31]. The placental labyrinthine layer, as the core region for exchange across placenta, is composed of the maternal blood sinuses and the fetal capillary network. Our H&E staining analysis revealed significant pathological alterations in the labyrinthine layer in the Model group compared to the Control group. These included disruption of the intermediate barrier, irregular maternal sinus morphology with associated pyknotic nuclei. Furthermore, the vascular density was significantly reduced under low-magnification microscopy. However, after EA intervention, all the above pathological changes were significantly improved, and the vascular density increased. It is suggested that EA at the "ST36" acupoints in maternal rats can alleviate insufficient placental angiogenesis in this model.

While these findings demonstrated that EA ameliorated PNE-induced IUGR and enhanced placental perfusion and vascular density, the underlying molecular mechanisms remained to be determined. Bulk RNA-seq technology is a method that can comprehensively and rapidly assess the transcriptional dynamics of biological tissues under different conditions. This technology is used to analyze DEG profiles and is widely applied in pregnancy disease research. Therefore, we conducted RNA-Seq analysis on the placental tissues of rat fetuses in different groups. The results showed that PGF was significantly downregulated in the Model group compared to the Control group but was significantly upregulated in the EA group compared to the Model group, suggesting that EA may exert a protective effect against IUGR by up-regulating PGF. We next performed KEGG pathway enrichment analysis on the DEGs. The results revealed that the DEGs of Model vs. Control and EA vs. Model groups were significantly enriched in signaling pathways such as PI3K/AKT, suggesting that the effect of EA on the rat placenta may be related to the activation of the PI3K/AKT signaling pathway.

It is worth noting that the placental vascular development mainly depends on the vascular endothelial growth factor family [40]. The members of this family mainly include VEGF-A, VEGF-B, VEGF-C, VEGF-D and PGF. Among them, the expression of PGF is closely related to IUGR. Multiple clinical studies have confirmed that compared with normal pregnancy, the expression of PGF in the placenta of patients with IUGR is significantly decreased [41, 42], and the degree of reduction is positively correlated with the severity of the disease [43]. As a member of the VEGF family, PGF is a pro-angiogenic protein [44], mainly binding to the VEGFR-1 to exert biological effects [45]. Studies have shown that knockdown of PGF or VEGFR-1 can inhibit the proliferation and migration of endothelial cells and delay angiogenesis, wound healing in a wound injury repair mouse model [46]. After binding to its receptor, PGF can activate a variety of intracellular signaling molecules, including PI3K, AKT, extracellular signal-regulated kinase 1/2 (ERK1/2), and p38 mitogen-activated protein kinase (MAPK) [47]. Among them, the PI3K/AKT pathway is the main pathway that controls cell proliferation and growth and plays an important role in angiogenesis [48]. As a key upstream factor of the pathway, after Class I PI3K is activated, it catalyzes the conversion of PIP2 into the second messenger PIP3. PIP3 then recruits and fully activates the core-effect kinase AKT. Activated AKT synergically promotes the proliferation, migration and survival of vascular endothelial cells and inhibits their apoptosis by phosphorylating multiple downstream target proteins, thereby driving the formation and maintenance of the placental vascular network, which is crucial for placental development and function. Studies in intestinal microvascular endothelial cells have confirmed [49] that PGF effectively promotes the migration of endothelial cells and the ability of lumen formation through the PI3K/AKT signaling pathway. These two behaviors are the key steps of angiogenesis. This indicates that PGF regulates endothelial cell function through the PI3K/AKT pathway, which is one of the core mechanisms by which it exerts its pro-angiogenic effect.

The RNA-Seq data showed that although the expression trends of VEGFR-1, the catalytic subunit gene of PI3K (PIK3CA), and AKT1 were consistent with those of PGF, they did not reach statistical significance. To verify the existence of this potential signaling axis, we expanded the sample size and conducted a targeted analysis using qRT-PCR and Western Blotting. The results indicated that EA not only significantly upregulated the expression of PGF but also synergistically activated its receptor VEGFR-1 and downstream PI3K/AKT signaling molecules. Therefore, EA may exert a protective effect by up-regulating the expression of PGF and VEGFR-1, thereby activating the PI3K/AKT signaling pathway, ultimately increasing placental angiogenesis. It should be noted that future studies employing pathway-specific inhibition are needed to provide conclusive proof of causality. Addressing this aspect will further solidify the mechanistic framework and lead to a more comprehensive understanding of EA’s protective role.

Moreover, our placental RNA-seq data intriguingly revealed the regulation of glucocorticoid (GC) homeostasis in our model: expression of 11β-hydroxysteroid dehydrogenase 2 (11β-HSD2) was significantly downregulated in the Model group but restored in the EA group, suggesting that EA may ameliorate GC dysregulation. In the pathological environment of IUGR, the imbalance of GC homeostasis is regarded as one of the key mechanisms that impairs placental vascular development [50]. Placental 11β-HSD2 plays a central role by inactivating GC to maintain appropriate intrauterine levels. However, studies have found that nicotine inhibits the expression of placental 11β-HSD2 [51], thereby leading to an abnormally elevated level of active GC in trophoblast cells [52]. Excessive GC can directly act on the fetal hypothalamic–pituitary–adrenal axis to inhibit its growth and development [53], and it can also damage the vascular structure of the placenta and affect its function, resulting in IUGR in the offspring. Consistent with this, studies in mice exposed to dexamethasone injection have demonstrated significantly reduced maximum placental diameter, junctional and labyrinthine volumes, and vascular density, along with decreased mRNA expression of VEGFA, VEGFC, and PGF [54]. While our data establishes EA’s dual regulation of GC homeostasis and PGF signaling, the causal relationship between these pathways remains unresolved. Specifically, it is unclear whether following EA, GC reduction directly upregulates PGF or whether 11β-HSD2 activity is a prerequisite for PGF induction. Future studies should clarify the functional hierarchy between 11β-HSD2 and PGF signaling in mediating EA’ s protective effects.

## Conclusion

In conclusion, electroacupuncture at the ST36 in pregnant rats ameliorated PNE-induced IUGR and improved placental perfusion. These benefits may be mediated by upregulation of placental PGF expression and subsequent activation of the PI3K/AKT pathway, which promotes placental angiogenesis, thereby enhancing perfusion and potentially improving pregnancy outcomes.

## Data Availability

The datasets used and/or analysed during the current study are available from the corresponding author on reasonable request.

## Abbreviations

IUGR: Fetal intrauterine growth restriction
PNE: Prenatal nicotine exposure
EA: Electroacupuncture
WB: Western blotting
H&E: Hematology and eosin
RNA-seq: RNA sequencing
PGF: Placental growth factor
VEGFR-1: Vascular endothelial growth factor receptor 1
PI3K: Phosphatidylinositol 3-kinase
AKT: Protein kinase B
11β-HSD2: 11β-Hydroxysteroid dehydrogenase 2
GC: Glucocorticoid
ERK1/2: Extracellular signal-regulated kinase 1/2
MAPK: Mitogen-activated protein kinase
BP: Biological process
CC: Cell composition
MF: Molecular function
GO: Gene ontology
KEGG: Kyoto encyclopedia of genes and genomes
DEGs: Differentially expressed genes
PI: Pulsatility index
RI: Resistance index
S/D: Systolic-diastolic ratio
